# The genetic basis for panicle trait variation in switchgrass (*Panicum virgatum*)

**DOI:** 10.1007/s00122-022-04096-x

**Published:** 2022-07-02

**Authors:** Li Zhang, Alice MacQueen, Xiaoyu Weng, Kathrine D. Behrman, Jason Bonnette, John L. Reilley, Francis M. Rouquette, Philip A. Fay, Yanqi Wu, Felix B. Fritschi, Robert B. Mitchell, David B. Lowry, Arvid R. Boe, Thomas E. Juenger

**Affiliations:** 1 Department of Integrative Biology, University of Texas at Austin, Austin, TX 78712 USA; 2 Kika de la Garza Plant Materials Center, National Resources Conservation Service, US Department of Agriculture, Kingsville, TX 78363 USA; 3 Texas A&M AgriLife Research and Extension Center, Texas A&M University, Overton, TX 75684 USA; 4 Grassland, Soil and Water Research Laboratory, Agricultural Research Service, US Department of Agriculture, Temple, TX 76502 USA; 5 Department of Plant and Soil Sciences, Oklahoma State University, Stillwater, OK 74078 USA; 6 Division of Plant Sciences, University of Missouri, Columbia, MO 65211 USA; 7 Wheat, Sorghum, and Forage Research Unit, Agricultural Research Service, US Department of Agriculture, University of Nebraska–Lincoln, Lincoln, NE 68583 USA; 8 Department of Plant Biology and DOE Great Lakes Bioenergy Research Center, Michigan State University, East Lansing, MI 48824 USA; 9 Departmentof Agronomy, Horticulture & Plant Science, South Dakota State University, Brookings, SD 57007 USA

## Abstract

**Key message:**

We investigate the genetic basis of panicle architecture in switchgrass in two mapping populations across a latitudinal gradient, and find many stable, repeatable genetic effects and limited genetic interactions with the environment.

**Abstract:**

Grass species exhibit large diversity in panicle architecture influenced by genes, the environment, and their interaction. The genetic study of panicle architecture in perennial grasses is limited. In this study, we evaluate the genetic basis of panicle architecture including panicle length, primary branching number, and secondary branching number in an outcrossed switchgrass QTL population grown across ten field sites in the central USA through multi-environment mixed QTL analysis. We also evaluate genetic effects in a diversity panel of switchgrass grown at three of the ten field sites using genome-wide association (GWAS) and multivariate adaptive shrinkage. Furthermore, we search for candidate genes underlying panicle traits in both of these independent mapping populations. Overall, 18 QTL were detected in the QTL mapping population for the three panicle traits, and 146 unlinked genomic regions in the diversity panel affected one or more panicle trait. Twelve of the QTL exhibited consistent effects (i.e., no QTL by environment interactions or no QTL × E), and most (four of six) of the effects with QTL × E exhibited site-specific effects. Most (59.3%) significant partially linked diversity panel SNPs had significant effects in all panicle traits and all field sites and showed pervasive pleiotropy and limited environment interactions. Panicle QTL co-localized with significant SNPs found using GWAS, providing additional power to distinguish between true and false associations in the diversity panel.

**Supplementary Information:**

The online version contains supplementary material available at 10.1007/s00122-022-04096-x.

## Introduction

As the bearers of grain, grass panicles (or inflorescences) have been targets of selection for thousands of years (Doust 2007). There is enormous diversity in panicle architecture within and among grass species (Coen and Nugent 1994). Panicle architecture is a critical determinant of interspecies differences in plant morphology and life history, which is often measured as variation in panicle length, branching structure (number, length, and pattern), and flower number and size borne on each branch type. Simple panicles may have only primary branches, while complex panicles can possess many secondary and tertiary branches (Bommert and Whipple 2018; Glemin and Bataillon 2009). In wild grasses, branching pattern plays an important role in wind pollination and affects the number and size of seeds, which ultimately influences seed yield and plant fitness (Brown et al. 2006; Friedman and Harder 2004). In domesticated species, there is a direct association between panicle architecture and seed productivity (Brown et al. 2006; Crowell et al. 2016; Wang and Li 2005). Analysis of the phylogenetic distribution of panicle variation in the grasses suggests that different panicle architectures have arisen independently many times, and homoplasy across the grass phylogeny has obscured the mechanisms of panicle diversity (Doust and Kellogg 2002; Kellogg 2000). These traits likely evolve in response to natural selection mediated by aspects of wind pollination (Friedman and Harder 2004) and environmental variation such as light (Vogler et al. 1999), drought (Mal and Lovett-Doust 2005; Caruso 2006), nutrient availability (Dorken and Barrett 2004), and intraspecific competition (Wolfe and Mazer 2005). Given the importance of inflorescence architecture to the fitness of wild species and the productivity of domesticated species, it is of great interest to understand genetic variation in panicle architecture.

Plant reproductive components (inflorescences, flowers, seeds) often exhibit phenotypic plasticity in response to the environment, perhaps related to evolve reproductive allocation trade-offs (Bazzaz and Grace 1997). In domesticated crops, selection has likely favored stability in grain yield possibly through reducing genetic variation in plasticity or favoring certain allocation trade-offs (Gage et al. 2017). Genetic variation in phenotypic plasticity in response to the environment is better known as genotype-by-environment interactions (GxE) (Des Marais et al. 2013). As such, insights in the genetics of plasticity or stability may be helpful for understanding the evolution of panicle traits or the impact of artificial selection in domestication. Quantitative genetic studies of GxE in many plant species (e.g., maize, rice) have identified important quantitative trait loci (QTL) impacting many panicle traits (Adriani et al. 2016; Doust et al. 2005; Leng et al. 2017; Liu et al. 2008; Miura et al. 2010). For example, Doust et al. (2005) detected 14 QTL for four inflorescence traits under two trials with high and low planting density underlying divergence between foxtail and green millet. They further found significant GxE for primary branch number and bristle number per primary branch with joint QTL analysis. GxE is common in QTL studies and identifying GxE and the pattern of interactions has implications for the role of genetic architecture underlying phenotypic traits and their response to selection. Genome-wide association studies (GWAS) and studies of GxE of panicle architecture have been common in various crops (Zhao et al. 2016; Bai et al. 2016; Liu et al. 2018; Ta et al. 2018; Thapa et al. 2021; Zhong et al. 2021; Wang et al. 2021). For example, Wang et al. (2021) performed association mapping of panicle morphology-related traits in the sorghum mini core panel measured in multiple environments. They identified several loci that were related to panicle traits and suggested a number of candidate genes that resided in the loci. Their study also suggested that GWAS study of GxE may facilitate the molecular identification of panicle morphology-related genes and the enhancement of yield and adaptation in sorghum. Identifying the genetic basis of panicle traits in additional species, and evaluating the evidence for GxE using both quantitative studies and GWAS, will increase our understanding of the genetic regions responsible for panicle architecture.

Switchgrass (*Panicum virgatum* L.) has been championed as a potential biofuel crop since its selection by the US Department of Energy (US DOE) as a model grass species for bioenergy in the early 1990s (Hohenstein and Wright 1994; McLaughlin 1993). The potential for high biomass production on marginal land, adaptation to a wide range of environments, and ecosystem service such as carbon sequestration, water flow management, and erosion control, make switchgrass an excellent candidate for meeting bioenergy needs (Mitchell et al. 2012; Robertson et al. 2017). Switchgrass is a warm-season C4 perennial grass native to the North America, with a range that extends from the eastern seaboard west to the Rocky Mountains and from southern Canada south to the Texas Coastal Plain and Northern Mexico (Casler 2007; Hopkins 1995). Two major distinctive ecotypes, northern upland and southern lowland ecotypes, have been classified in the past based on morphology and habit preference (Porter Jr 1966). A recent study based on a resequenced switchgrass diversity panel defined a third coastal ecotype, which is broadly sympatric with the lowland ecotype but possesses upland leaf characters and lowland plant morphotype (Lovell et al. 2021).

Information on panicle morphology is limited in switchgrass, although panicle length differences have been reported between switchgrass ecotypes and cultivars (Porter Jr 1966; Price 2014; Van Esbroeck 2003). We hypothesize that panicle evolution in switchgrass may be related to selection on aspects of mating system and degree of investment in vegetative versus sexual reproduction, especially in the context of seedling establishment in differing habitats. For example, lowland switchgrass has a restricted bunch grass growth form and occurs primarily in patchy distributions along riparian areas. In contrast, upland switchgrass has a rhizomatous spreading growth form that occurs in many prairie habitats. Pattern of pollen dispersal across patches, or aspects of seed establishment (e.g. seed size/number trade-offs or disturbance regimes) likely differ in these habitats and may have driven divergence in panicle form. Panicle morphology and its relationship to seed quality may be important targets of selection and breeding, as consistent seed production will be critical to meet the demands for large-scale biofuel production (Das and Taliaferro 2009; Vogel 2000).

In this study, we evaluated the genetic architecture of switchgrass panicle traits in two mapping populations: a pseudo-F2 mapping population (hereafter, ‘four-way’) grown across ten field sites (or common gardens) in the central USA and a natural population of switchgrass (hereafter, ‘diversity panel’) grown at three of the ten sites. We assessed three panicle traits for each population at the end of their respective growing seasons: panicle length (PL), primary branching number (PBN) per panicle, and secondary branching number (SBN). For these phenotypes, we assessed (1) the genetic architecture underlying the trait, (2) the sensitivity of QTL and their effects across different environments from the four-way, (3) the single nucleotide polymorphism (SNP) effects on the traits in the three common gardens from the diversity panel, and (4) the candidate genes that were found for both two populations potentially involved in the regulation of panicle architecture in switchgrass.

## Materials and methods

### Field experiment and phenotyping of the four-way

The details of the creation of the four-way population are described in Milano et al. (2016). Briefly, the grandparents of the mapping population were derived from highly divergent southern lowland and northern upland ecotypes. The population was developed by initial crosses between AP13 (A) × DAC6 (B) and WBC3 (C) × VS16 (D). AP13 and WBC3 are genotypes clonally derived from an individual selected from the lowland cultivar ‘Alamo’ (southern Texas accession) and an individual from naturally occurring population ‘West Bee Cave’ (central Texas accession), respectively. DAC6 and VS16 are genotypes clonally derived from individuals selected from the upland cultivars ‘Dacotah’ and ‘Summer’ (both northern upland accessions), respectively. The F_1_ hybrids of each of those crosses were then intercrossed reciprocally to produce the four-way outbred mapping population.

**Fig. 1:**
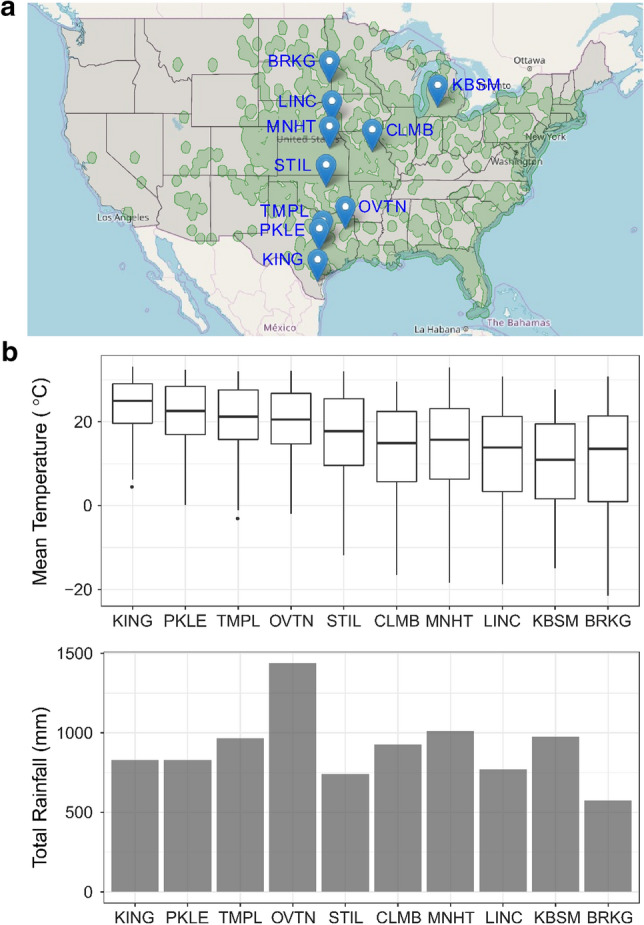
The geographic location and climate at the ten field sites. **a** The ten sites across the latitudinal gradients from southern Texas to South Dakota. The experimental sites in this study span much of the natural range of switchgrass (the green layer with buffered points). **b** The mean temperature and annual rainfall of the ten sites for the study year in 2016 for the four-way QTL population (ordered from south to north)

The grandparents, F_1_ hybrid parents, and the F_2_ progeny were propagated by dividing plants manually to produce 10 clones, each of which was maintained in a 3.8-L pot at the Brackenridge Field Laboratory, Austin, TX in 2013–2015. One replicate of each of the mapping progeny genotypes (i.e., 380 core genotypes), along with multiple replicates of grandparents and F_1_ parents, was transplanted from May to July of 2015 at 10 field sites. The 10 field sites cover 17 degrees of latitude from South Texas to South Dakota (Fig. 1A). Detailed information of the 10 field sites, including latitude, longitude, and soil type, is provided in Table 1. The annual mean temperature at the 10 sites in 2016 ranged from 10.4 in the north to 20.7 °C in the south, and the total rainfall varied from 574 to 1440 mm (Fig. 1B, data are from local weather station or from NOAA if local weather data are not available; the weather station or NOAA link is included in Table 1). To control weeds, each field site was covered with one layer of weed barrier cloth (Dewitt, Sikeston, MO). Holes were cut into the weed cloth in a honeycomb fashion. Plants were randomized into the holes, with each plant having four nearest neighbors each located 1.56 m away from each other. A row of border plants was planted at every edge position of the field to minimize edge effects. The border plants were derived from rhizome plugs obtained from an approximately 10-year-old stand of Alamo switchgrass. Plants were well watered in the field during the summer of 2015 to facilitate establishment and all phenotypes were collected in 2016.

**Table 1:** The latitude, longitude, site code, soil texture, and source of weather data for the ten experimental fields of the four-way and the three overlapping sites (marked as *) of the diversity panel in the study

Field site	Site code	Latitude	Longitude	Soil texture	Weather data source
Brookings, SD	BRKG	44.307	− 96.67	Clay loam	https://www.ncdc.noaa.gov/cdo-web/results
Hickory Corners, MI	KBSM*	42.42	− 85.37	Loam	https://lter.kbs.msu.edu/datatables/7
Lincoln, NE	LINC	41.154	− 96.42	Loam	https://www.ncdc.noaa.gov/cdo-web/results
Manhattan, KS	MNHT	39.141	− 96.64	Sandy loam	mesonet.k-state.edu/weather/historical
Columbia, MO	CLMB*	38.897	− 92.22	Loam	http://agebb.missouri.edu/weather/history/index.asp?station_prefix=bfd
Stillwater, OK	STIL	35.991	− 97.05	Sandy loam	https://www.mesonet.org/index.php/weather/local/perk
Overton, TX	OVTN	32.303	− 94.98	Sandy loam	https://www.ncdc.noaa.gov/cdo-web/results
Temple, TX	TMPL	31.043	− 97.35	Clay	https://www.ars.usda.gov/plains-area/temple-tx/grassland-soil-and-water-research-laboratory/docs/temple-climatic-data/
Austin, TX	PKLE*	30.384	− 97.73	Clay	https://www.ncdc.noaa.gov/cdo-web/results
Kingsville, TX	KING	27.55	− 97.88	Sandy clay loam	https://www.ncdc.noaa.gov/cdo-web/results

**Fig. 2:**
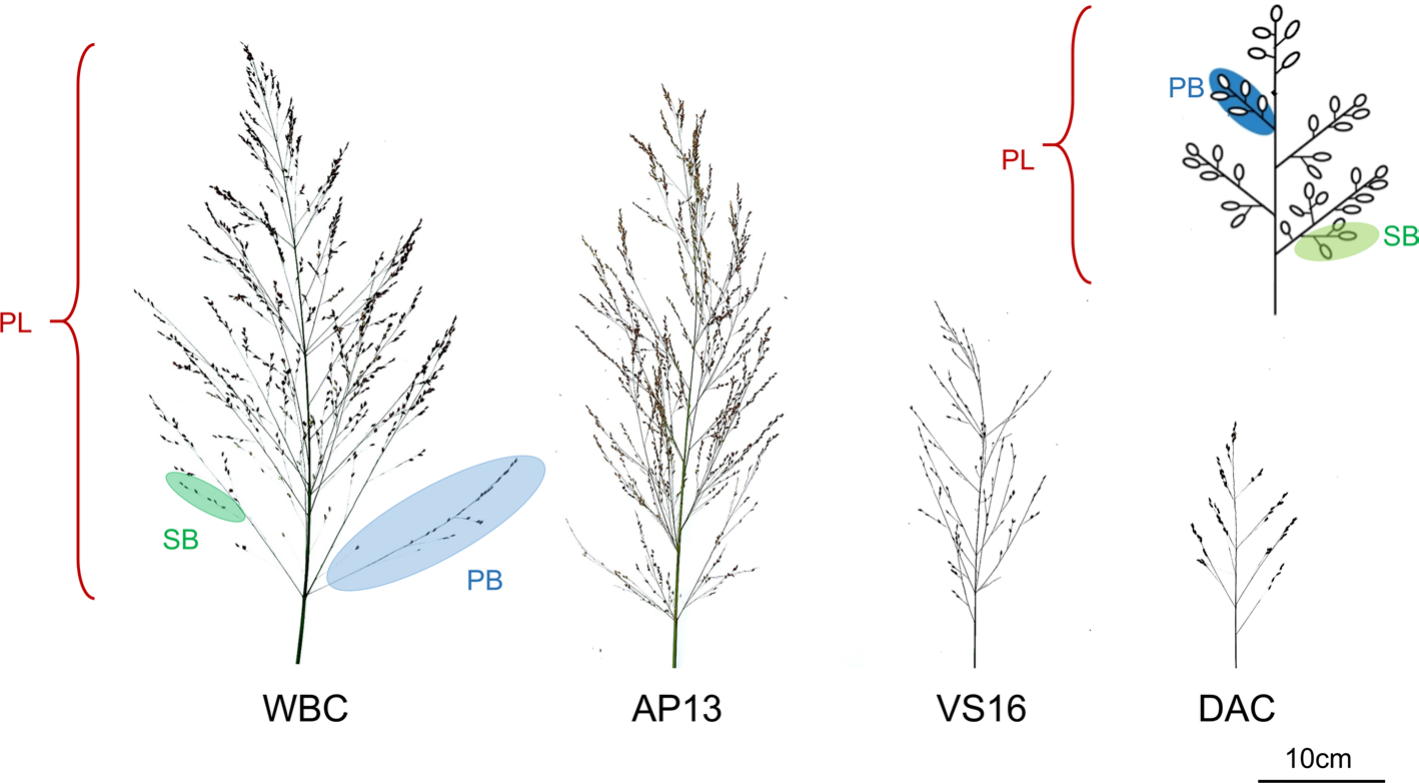
Representative panicles of switchgrass from the four grandparents (WBC, AP13, VS16, and DAC) of the four-way. PL is the panicle length (in mm), PBN is the primary branching number, and SBN is the secondary branching number

Three panicles were randomly sampled from the tallest tiller of each plant at full maturity. Panicle length (PL in mm), primary branching number (PBN), and secondary branching number (SBN) were assessed at the end of the 2016 growing season. A diagram depicting these phenotypes is presented in Fig. 2, with representative images of panicles from the four grandparents. PL was measured on the primary panicle from the base of the first primary branch to the top of the panicle. PBN was counted as the total number of branches along the primary rachis. Due to the numerous secondary branches in switchgrass, SBN in our study referred to the total number of secondary branches on the lowest primary branch of the panicle (Fig. 2). In total, over 10,000 separate panicle morphology measurements were collected from the four-way population. The phenotypic data (i.e., average values) for each genotype at each field site are provided in Supplemental Table S1.

### Genotyping and multi-environment QTL modeling of the four-way

Details on the genetic map construction can be accessed on https://datadryad.org/stash/dataset/doi:10.5061/dryad.ghx3ffbjv (Lovell et al. 2020) and in Bragg et al. (2020). In brief, Illumina fragment paired end libraries from each of the four grandparents were aligned to the P. *virgatum* reference genome v5 and used for single-nucleotide polymorphism (SNP) calling. Then, a kmer-based approach was used to capture multiple variant and distinguish each grandparent when genotyping the progeny. The resulting genotype matrix was polished via sliding windows across the physical V5 switchgrass genome position and markers were re-ordered within linkage groups (Lowry et al. 2019; Lovell et al. 2020). Genotypes for progeny were based on grandparental haplotypes and thus are fully informative. The genetic map spans 750 recombinant 4-way progeny genotyped at 4700 markers. For computational efficiency in GxE analysis, the genetic map was reduced to 738 markers, with an average distance of 2 cM between markers.

We estimated quantitative genetic variation for the panicle traits of the four-way using marker-based realized relationship matrices and linear mixed models implemented in the Sommer package (Covarrubias-Pazaran 2016) in R Core Team (2020). Since additive genetic variance and dominance are not orthogonal in a full-sib family like the four-way, it was not feasible to cleanly partition additive from nonadditive components of variance (Hill 2013). As such, we report our estimates from the kinship matrix as genetic variance (*V*g), and our heritabilities as broad-sense heritability (*H*^2^), which was calculated as *V*g/*V*p, where *V*p is the total phenotypic variance. We calculated the *H*^2^ for each trait at each field site. In addition, we tested for GxE for each trait using the same mixed model approach (Covarrubias-Pazaran 2016, https://cran.r-project.org/web/packages/sommer/vignettes/v4.sommer.gxe.pdf, last accessed in Aug, 2021). Here, we tested whether *V*g differed by site for each panicle trait. Briefly, we used a likelihood-ratio test to compete between a base main effect model to an unstructured model allowing for GxE across sites. The main effect model assumes that a single *V*g parameter plus a fixed effect for environment is enough to predict the genotype effect in all locations of interest. We compared this base model to an unstructured model that estimates a unique genetic variance and covariance within and across environments (a 10 × 10 unstructured variance–covariance matrix in our study). Significance of the likelihood-ratio test for GxE was assessed at the level of *α* = 0.05.

Details of the mapping scheme and application in the outbred four-way population are described in Malosetti et al. (2013) and Lowry et al. (2019). In brief, ‘single trait under multiple environments’ QTL mapping for each panicle trait in the cross-pollinated (CP) family was implemented in VSN International (2019). The QTL approach with CP family resulted in four possible QTL alleles designated A and B corresponding to marker alleles of the first pair of grandparents (AP13 × DAC) and QTL alleles C and D corresponding to marker alleles of the second pair of grandparents (WBC × VS16). A multienvironment mixed model was fit for each trait as shown in Eq. 1:

1 $${\text{trait}} = \mu + E + \sum {\text{QTL}} + \sum \left( {{\text{QTL}} \times E} \right) + e$$

where *μ* is the population mean; *E* represents the environment effect; $\sum \mathrm{QTL}=\sum \left({a}^{a1}+{a}^{a2}+{a}^{d}\right)$, denoting the total effect from the additive effect from the first grandparent (i.e., the difference between A and B alleles, ${a}^{a1}$, the second grandparent (i.e., the difference between C and D alleles, ${a}^{a2}$, and the dominance effect (i.e., the intralocus interaction, ${a}^{d}$; $\sum \left( {{\text{QTL}} \times E} \right)$ represents the QTL × environment interactions; and *e* represents the error term that was modeled by an unstructured variance–covariance matrix. The unstructured model was used to specify the data structure in the genome-wide QTL scan of simple interval mapping (SIM) and composite interval mapping (CIM). A backward selection procedure was used to retain significant fixed terms (*p* < 0.05) after three consecutive runs of CIM to confirm stability of QTL. The QTL with highest LOD peaks were considered as the most significant QTL, and the flanking markers associated with 1.5 LOD drop around the most significant QTL were considered as confidence interval for the QTL peaks.

### Genome-wide association and multivariate adaptive shrinkage in the switchgrass diversity panel

The formation and resequencing of the switchgrass diversity panel has been described previously (Lovell et al. 2021). Briefly, hundreds of tetraploid switchgrass plants were resequenced, and these genotypes were clonally replicated and planted at multiple common gardens spanning a latitudinal gradient across the continental USA. We phenotyped panicle length (PL in mm), primary branch number (PBN), secondary branch number (SBN) as above for three panicles cut from each plant at full maturity at the end of the 2019 growing season, in a subset of genotyped individuals and common gardens. We phenotyped 382 genotyped individuals that had clones present at each of three common garden locations (Austin, TX or PKLE; Columbia, MO or CLMB; and Hickory Corners, MI or KBSM), which overlapped with the three sites in the four-way and cross a large latitude of Central US. We took three measurements per individual at each field site, then found phenotypic BLUPs (Best Linear Unbiased Predictions) for each genotype in ASReml-R (Butler et al. 2017) using the model: trait ~ genotype + error, where genotype was a random factor and both genotype and error were fitted as identity variance models (~ idv()). The phenotypic data (raw values and phenotypic BLUPs) for each genotype at each field site for the diversity panel are provided in Table S1.

We analyzed SNP (single nucleotide polymorphism) effects on three panicle traits in three common gardens using multivariate adaptive shrinkage (mash), using effect estimates from univariate genome-wide association studies (GWAS). GWAS were conducted using the bigsnpr R package (Privé et al. 2018), which performs fast statistical analysis of large SNP arrays encoded as matrices, and which implements the current best practices in human genetics for principal component analysis of population genetic data (Privé et al. 2020). Only SNPs with < 20% missing data and minor allele frequencies > 0.05 at all three gardens were used in univariate GWAS, resulting in 18.7 M SNPs retained for the analysis. We used singular value decomposition (SVD) on all 18.7 M SNPs for all 382 genotyped individuals to create 15 genetic principal components (PCs) for population structure correction using the snp_autoSVD() function in bigsnpr. To choose the number of PCs that best controlled for population structure and reduced genomic inflation, we ran univariate linear regressions for each combination of phenotype and common garden including the range of 0–15 PCs as covariates, then selected either the smallest number of PCs that made *λ*_GC_, the genomic inflation factor, less than 1.05, or else selected the number of PCs that minimized *λ*_GC_ when the first criterion could not be met (Supplemental Table S2). In practice, because the PCs are orthogonal by definition, GWAS results are not sensitive to the number of PCs used, as long as a sufficient number of PCs are included to capture true population structure effects (Price et al. 2006). Our first two PCs accounted for population structure, and our third and fourth the effects of ecotype within genetic subpopulations; further PCs captured additional structure between the genetic subpopulations not tied to known phenotypic differences, suggesting at least four PCs should be used (Figure S1). Our univariate GWAS all used at least four PCs to account for population structure, and all had *λ*_GC_ < 1.043; our nine univariate GWAS (3 traits by 3 locations) had strong associations and appeared free of obvious population structure confounding issues (Figure S2).

We then ran mash on the effect estimates and standard errors generated from univariate GWAS, following mash documentation (Urbut et al. 2019): first, 100 K SNPs unlinked at an *r*^2^ of 0.2 were used as a ‘random’ set to learn the background correlation structure; second, 5 K SNPs with the maximum − log10p values in any of the univariate GWAS were used to construct data-driven covariance matrices; third, the random set was used to fit the mashr model; fourth, posterior summaries using the model fit on the random set were computed on all 18.7 M SNPs. We generated six data-driven matrices in the mash run, five (denoted ED_PCA_1 through ED_PCA_5) produced by singular value decomposition (SVD) of an overall matrix, denoted ‘ED_tPCA.’ The ED prefix refers to the extreme deconvolution algorithm used by mash to derive the data-driven matrices. We determined which SNPs had evidence of significant phenotypic effects using local false sign rates (lfsr), which are analogous to false discovery rates but more conservative (in that they also reflect the uncertainty in the estimation of the sign of the effect) (Urbut et al. 2019). These lfsr were condition-specific; for an overall measure of significance for each SNP, we used the log10 (Bayes Factor) computed by mash, which measures the overall significance of a SNP on the trait effects included in mash.

### Enrichment tests to find candidate genes in both mapping populations

To determine if SNPs with significant trait effects on panicles in our diversity panel (assessed using mash) were enriched in panicle QTL intervals in our four-way mapping population, we compared SNP enrichment in the QTL intervals to SNP enrichment of 1000 permutations of the QTL regions. First, to reduce enrichments due only to partially linked SNPs within a QTL region, the 18.7 M SNPs used in mash were clumped to keep only the most significant SNP in each LD block, using a linkage threshold of *r*^2^ < 0.2. Significance was assessed using the log10 (Bayes Factor). SNP clumping resulted in 2.7 M SNPs unlinked at an *r*^2^ of 0.2. Second, 1000 permutations of the QTL regions were created of the same size (in bp) of the 18 QTL found using the four-way mapping cross. For both the QTL intervals and these 1000 permutations, we assessed the number of QTL that had significant enrichments of mash SNPs in the top 1% percentile of the 2.7 M partially linked SNPs using hypergeometric tests. We also explored SNP effects in a completely unlinked set of SNPs, where the 18.7 M SNPs used in mash were clumped to keep only the most significant SNP in each LD block, using a linkage threshold of *r*^2^ < 0. SNP clumping at an *r*^2^ of 0 resulted in retention of 303 SNPs.

Third, we identified genes that were located both in the confidence intervals of the discovered QTL from the four-way and within 20 kb of the 6149 partially linked mash SNPs with log10 (Bayes Factor) > 1.3 from the diversity panel. Because these genes were identified in two independent mapping panels, we have increased confidence that these genes are involved in panicle architecture in switchgrass. We used the ‘pvdiv_table_topsnps’ function of the switchgrassGWAS R package (https://github.com/Alice-MacQueen/switchgrassGWAS) to find genes within 20 kb of mash SNPs, a distance consistent with a 50% linkage disequilibrium decay in this species (Grabowski et al. 2017; Lovell et al. 2021). These genes were compared with the rice (v7, accessed from phytozome https://phytozome-next.jgi.doe.gov/info/Osativa_v7_0) and Arabidopsis annotation databases (TAIR 10, accessed from phytozome https://phytozome-next.jgi.doe.gov/info/Athaliana_TAIR10) to further identify candidate genes with functional validation in panicle architecture, or bolt architecture after the transition to flowering, in other species (Bouché et al. 2016; Yao et al. 2018). The annotation file for switchgrass was accessed on JGI (Joint Genome Institute) Phytozome 13 website: https://njp-spin.jgi.doe.gov/.

## Results

### Phenotypic variation and heritability of the four-way

**Fig. 3:**
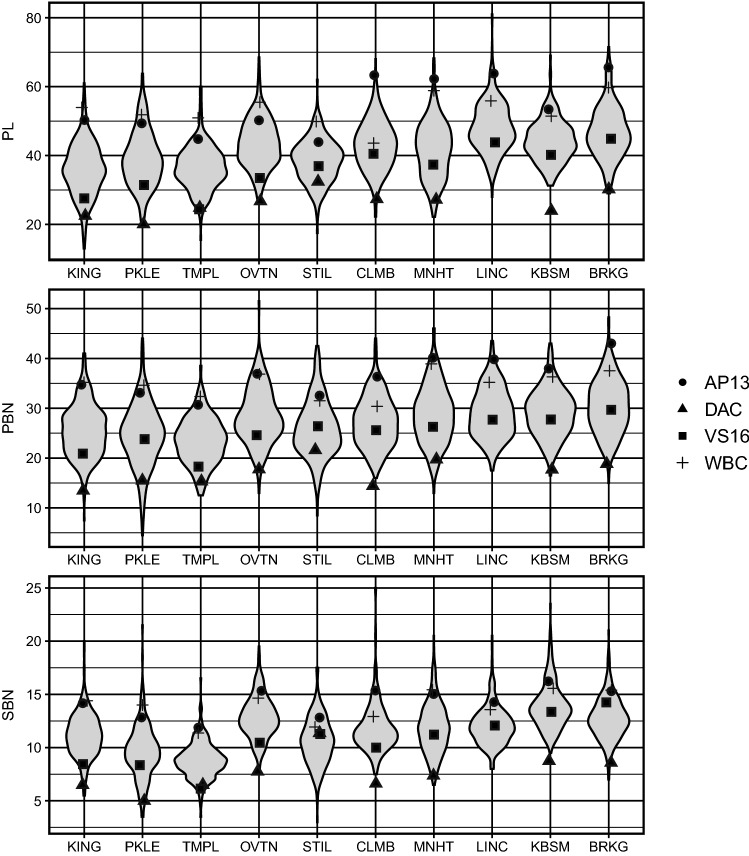
The phenotypic distribution of the F_2_ population, and the phenotypic means of the four grandparents (lowland AP13, WBC and upland DAC and VS16) of the four-way for panicle length (PL in mm), primary branching number (PBN), and secondary branching number (SBN) across the ten field sites (ordered from south to north)

Values for the three measured panicle traits increased in F_2_ individuals from the four-way as latitude of the common garden increased (Fig. 3). Each trait showed a continuous, unimodal distribution within sites, and transgressive behavior in the F_2_ generation. The lowland genotype F_0_ individuals, AP13 and WBC, always had larger values of panicle length (PL in mm), primary branching number (PBN), and secondary branching number (SBN) than the upland genotype F_0_ individuals, DAC and VS16 (Fig. 3). The phenotypic correlations between traits were generally positive but varied by site, ranging from 0.21 to 0.63 for phenotypic correlation (Table 2).

**Table 2:** The phenotypic correlation between panicle traits within sites

Sites		Phenotypic correlation			Sites		Phenotypic correlation		
		PL	PBN	SBN			PL	PBN	SBN
BRKG	PL	1	–	–	STIL	PL	1	–	–
	PBN	0.41	1	–		PBN	0.42	1	–
	SBN	0.42	0.39	1		SBN	0.42	0.57	1
KBSM	PL	1	–	–	OVTN	PL	1	–	–
	PBN	0.30	1	–		PBN	0.52	1	–
	SBN	0.42	0.54	1		SBN	0.59	0.58	1
LINC	PL	1	–	–	TMPL	PL	1	–	–
	PBN	0.43	1	–		PBN	0.35	1	–
	SBN	0.52	0.60	1		SBN	0.53	0.46	1
MNHT	PL	1	–	–	PKLE	PL	1	–	–
	PBN	0.56	1	–		PBN	0.27	1	–
	SBN	0.68	0.67	1		SBN	0.21	0.51	1
CLMB	PL	1	–	–	KING	PL	1	–	–
	PBN	0.36	1	–		PBN	0.52	1	–
	SBN	0.40	0.50	1		SBN	0.63	0.61	1

PL, panicle length, PBN, primary branching number, and SBN, secondary branching number for the four-way

The heritability (*H*^2^) for PL, PBN, and SBN varied by site and was typically moderate (0.2–0.5) or high (> 0.5) (Table 3). The *H*^2^ for PL ranged from 0.20 to 0.71, with an average of 0.46 and values greater than 0.50 at four of five northern sites. The *H*^2^ for PBN ranged between 0.45 and 0.66 for nine out of the ten sites, with Stillwater, OK (STIL) having low heritability (*H*^2^ = 0.20). The *H*^2^ for SBN ranged from 0.02 to 0.62, where Stillwater, OK (STIL) had *H*^*2*^ close to zero (*H*^2^ = 0.02), Columbia, MO (CLMB) had low heritability (*H*^2^ = 0.15), and four sites had heritability point estimates of approximately 0.50. Likelihood-ratio tests by comparing the model without GxE (i.e., main effect model) to the model with GxE (i.e., unstructured model) indicated that GxE existed for all the three panicle traits (*p* < 0.05). Thus, switchgrass exerted genetic control of panicle traits with environmental sensitivity.

**Table 3:** Broad-sense heritability (*H*^2^), and its one standard error (± 1 SE), for panicle length (PL), primary branching number (PBN), and secondary branching number (SBN) at each of the ten field sites (ordered from north to south) for the four-way

Sites/traits	PL	PBN	SBN
BRKG	0.55 ± 0.07	0.64 ± 0.06	0.40 ± 0.08
KBSM	0.71 ± 0.06	0.62 ± 0.07	0.56 ± 0.07
LINC	0.58 ± 0.07	0.65 ± 0.07	0.47 ± 0.08
MNHT	0.38 ± 0.08	0.66 ± 0.06	0.38 ± 0.08
CLMB	0.57 ± 0.07	0.64 ± 0.07	0.15 ± 0.08
STIL	0.20 ± 0.08	0.13 ± 0.08	0.02 ± 0.07
OVTN	0.44 ± 0.08	0.63 ± 0.06	0.62 ± 0.07
TMPL	0.49 ± 0.08	0.54 ± 0.07	0.30 ± 0.08
PKLE	0.24 ± 0.09	0.45 ± 0.09	0.29 ± 0.09
KING	0.47 ± 0.08	0.58 ± 0.07	0.48 ± 0.08

### Multi-environment mixed QTL model

**Fig. 4:**
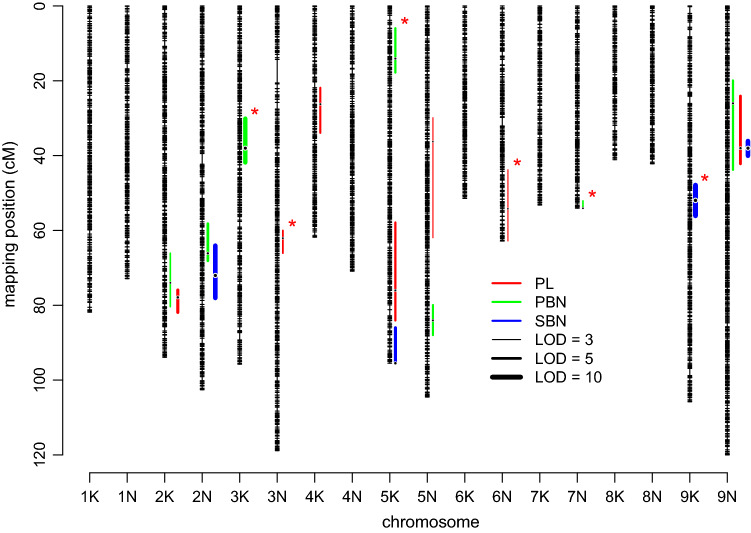
The summary of QTL and significant QTL-by-environment interaction (marked as * in red) identified from the four-way population for panicle length (PL in mm), primary branching number (PBN), and secondary branching number (SBN) (color figure online)

**Fig. 5:**
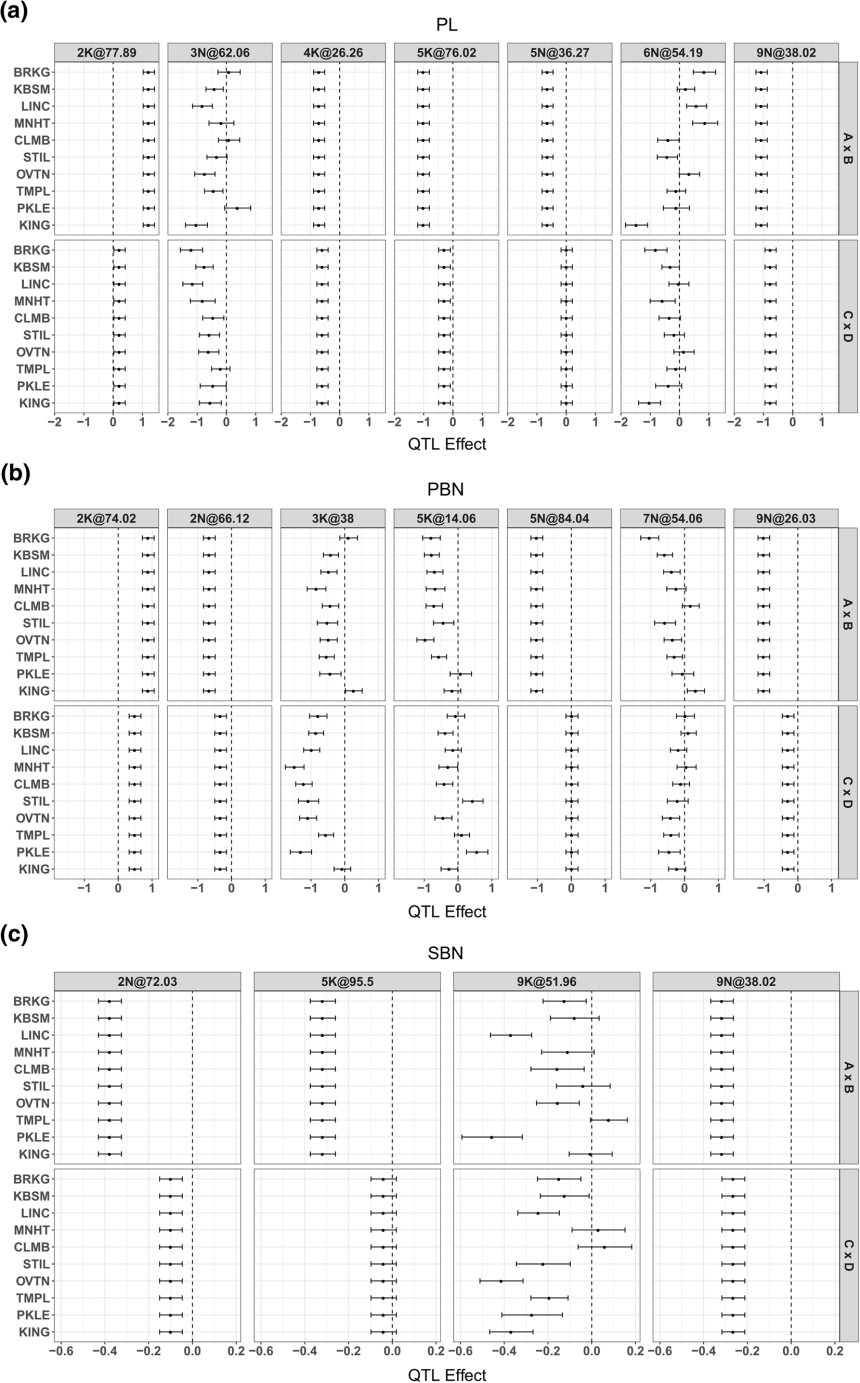
The additive effects of each QTL identified from the four-way for **a** panicle length (PL in mm), **b** primary branching number (PBN), and **c** secondary branching number (SBN) across geographic regions ordered by from south to north. Genstat reports all of the effects as equal when a QTL does not exhibit QTL × environment interaction

A total of 18 QTL were identified for panicle morphology traits using multi-environment mixed model analyses (Fig. 4, Table 4). Seven QTL were identified for PL, distributed across seven chromosomes. Among these, five QTL (2 K@77.89, 4 K@26.26, 5 K@76.02, 5 N@36.27, and 9 N@38.02) had consistent effects across field sites (Fig. 5a), while two QTL (3 N@62.06 and 6 N@54.19) showed interaction with the environment (QTL × E). The additive effects for QTL 3 N@62.06 changed in magnitude across geographic regions. QTL 6 N@54.19 (C × D cross) had the largest effects at the most northern and southern site and smaller effect at mid-latitude sites. There is also consistent directionality for these effects, that is, lowland alleles always make longer panicles (Fig. 5a, A × B and C × D crosses of 3 N@62.06, and C × D cross of 6 N@54.19). The A × B cross of QTL 6 N@54.19 had a trade-off pattern, with a sign change (aka. antagonistic pleiotropy) in allelic effects between three northern sites and the southernmost site.

**Table 4:** The identified QTL, along with their marker name (chromosome with physical distance in mega base pair), maximum LOD values, and flanking markers with a LOD drop of 1.5 for panicle morphology traits (PL: panicle length; PBN: number of primary branches; SBN: number of secondary branches) for the four-way. The presence of genotype by environmental interaction is marked as ‘Yes’ or ‘No’ in column QxE

Trait	QTL	MARKER	LOD	Left flanking marker	Right flanking_marker	QxE
PL	2 K@77.89	Chr02K_62.598826	6.18	Chr02K_60.739957	Chr02K_64.045891	No
PL	3 N@62.06	Chr03N_26.099983	4.29	Chr03N_24.521536	Chr03N_30.30364	Yes
PL	4 K@26.26	Chr04K_13.041487	4.66	Chr04K_9.916183	Chr04K_29.613196	No
PL	5 K@76.02	Chr05K_56.620419	4.82	Chr05K_44.678143	Chr05K_58.488157	No
PL	5 N@36.27	Chr05N_16.511689	3.63	Chr05N_11.735767	Chr05N_47.154718	No
PL	6 N@54.19	Chr06N_48.768076	3.56	Chr06N_43.871788	Chr06N_51.935176	Yes
PL	9 N@38.02	Chr09N_18.617122	5.82	Chr09N_10.880731	Chr09N_20.831824	No
PBN	2 K@74.02	Chr02K_59.503978	4.09	Chr02K_56.436103	Chr02K_63.664705	No
PBN	2 N@66.12	Chr02N_55.500715	5.52	Chr02N_50.387752	Chr02N_56.445418	No
PBN	3 K@38	Chr03K_17.77051	8.83	Chr03K_13.323286	Chr03K_20.786505	Yes
PBN	5 K@14.06	Chr05K_7.188103	4.90	Chr05K_4.388419	Chr05K_8.204815	Yes
PBN	5 N@84.04	Chr05N_64.047349	4.73	Chr05N_60.974614	Chr05N_65.990782	No
PBN	7 N@54.06	Chr07N_49.904749	4.17	Chr07N_49.035214	Chr07N_49.904749	Yes
PBN	9 N@26.03	Chr09N_12.531268	4.89	Chr09N_7.913256	Chr09N_21.588445	No
SBN	2 N@72.03	Chr02N_58.696003	9.46	Chr02N_54.556579	Chr02N_60.798034	No
SBN	5 K@95.5	Chr05K_60.232411	6.22	Chr05K_58.583292	Chr05K_60.232411	No
SBN	9 K@51.96	Chr09K_24.465322	10.37	Chr09K_19.959778	Chr09K_28.697896	Yes
SBN	9 N@38.02	Chr09N_18.617122	9.29	Chr09N_17.684245	Chr09N_19.333648	No

Seven QTL were identified for PBN distributed across seven chromosomes. Four QTL (2 K@74.02, 2 N@66.12, 5 N@84.04, and 9 N@26.03) had consistent effects across locations, while three QTL (3 K@38, 5 K@14.06, and 7 N@54.06) had QTL × E interactions, including both changes of magnitude (3 K@38 and 7 N@54.06) and direction (5 K@14.06) of the allelic effect across geographic regions (Fig. 5b). Four QTL were identified for SBN. Three QTL (2 N@72.03, 5 K@95.5, and 9 N@36.02) had consistent effects across locations, while there was a magnitude changing interactions for QTL 9 K@51.96 (Fig. 5c). Similarly, there is consistent directionality for the QTL effects with magnitude change lowland alleles often making more branches, including primary and secondary branches. We also observed that two QTL for PBN (2 K@74.02 and 9 N@26.03) co-localized with PL QTL on chromosome 2 K and 9 N, based on overlapping confidence intervals (Fig. 4). QTL 9 N@38.02 for SBN co-localized with the QTL of PL and PBN on chromosome 9 N (Fig. 4). The majority of QTL (12 of 18) did not show significant QTL x E interactions.

### Genome-wide association and multivariate adaptive shrinkage in the switchgrass diversity panel

**Fig. 6:**
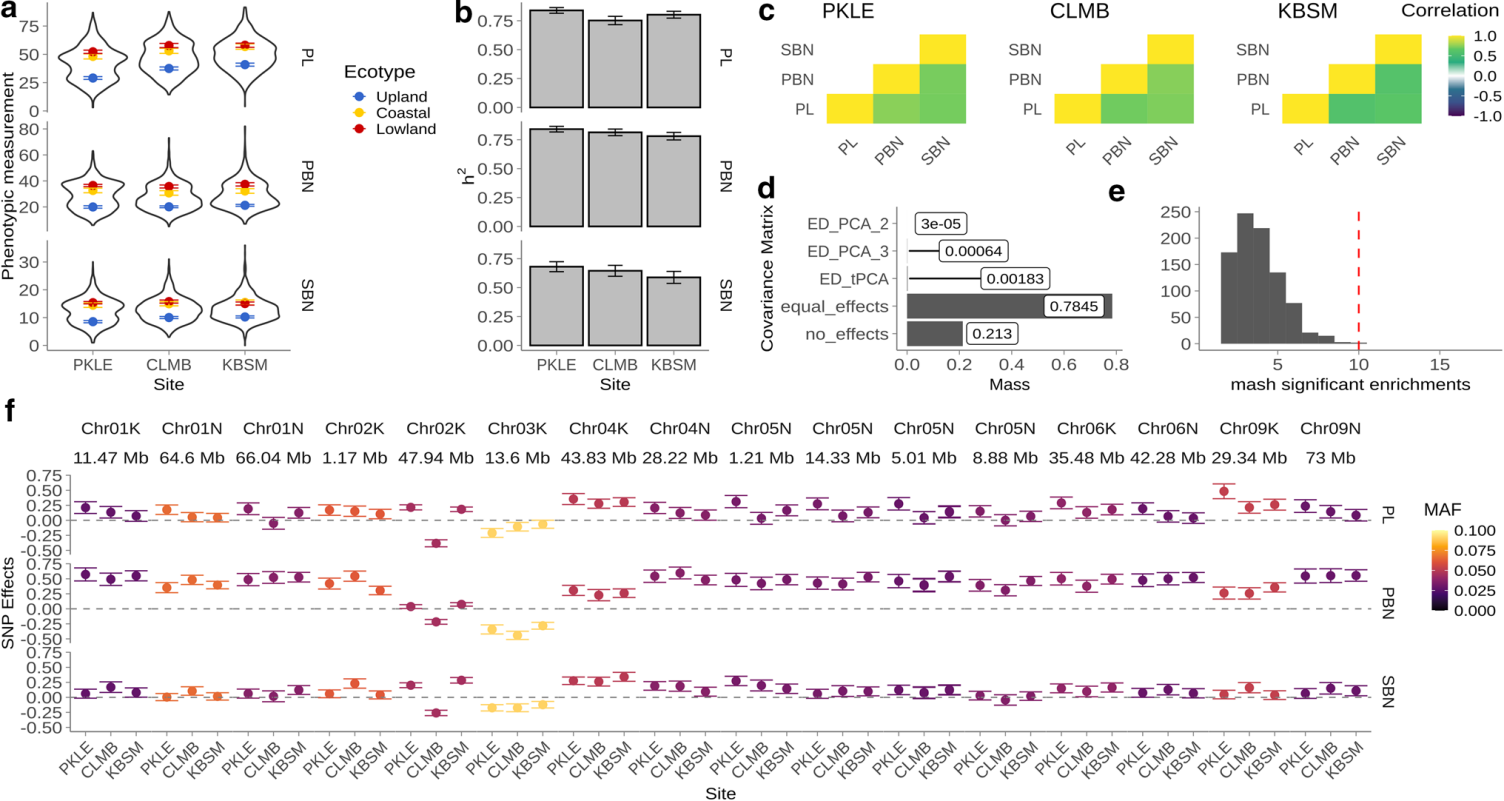
Phenotypic variation and genetic effects for panicle traits in a 382 individual diversity panel, analyzed at the three field sites (PKLE, CLMB, and KBSM in Fig. 1) using multivariate adaptive shrinkage, or mash. **a** The phenotypic distribution of the diversity panel (violin plots), and the phenotypic means for the three ecotypes (upland, lowland, and coastal) present in the panel (colored points and error bars) for panicle length (PL in mm), primary branching number (PBN), and secondary branching number (SBN) across the three field sites (ordered from south to north). **b** Narrow-sense heritability (*h*^2^) estimates for each panicle trait estimated separately at each site. Error bars represent two times the standard error. **c** Phenotypic correlations for each panicle trait within sites. **d** Posterior weights (mass) for SNPs with log10(Bayes Factor) > 1.3 on five covariance matrices. Blue indicates posterior weights for significant SNPs that are partially linked (*r*^2^ < 0.2; 6149 SNPs) and purple significant SNPs that are unlinked (*r*^2^ = 0; 146 SNPs). The all ones covariance matrix has positive covariances of one between all trait-site pairs, and the all zeroes matrix has covariances of zero between all trait-site pairs. Data-driven matrices are indicated by ‘ED’ and covariances between pairs of trait-sites can be seen in Figure S5. **e** Number of QTL with a significant enrichment of partially linked SNPs from mash is indicated by the dashed red line; number of permuted genomic regions with significant enrichment of the same SNPs is indicated by the histogram. **f** SNP effects estimated using mash for unlinked SNPs significant above a log10 (Bayes Factor) of 5. Points and error bars represent means and standard deviations estimated by mash, respectively; colors indicate the minor allele frequency for the SNP in the diversity panel (color figure online)

Three panicle traits were measured in 382 clonal propagates of a diversity panel grown in three common gardens. Trait values showed continuous distributions within sites which were bimodal for PL and PBN at all sites and for SBN at PKLE (Fig. 6a). These bimodal distributions were caused by unimodal trait distributions within ecotypes that had trait means and trait distributions which differed significantly between the upland ecotype and the coastal and lowland ecotypes at all sites (Figure S3); coastal and lowland ecotype trait means differed only for PBN, driven mostly by PBN differences between the Atlantic and Gulf genetic subpopulations (Figure S3). Narrow-sense heritabilities were high (> 0.5) for all traits at all sites (Fig. 6b), marginally higher in Texas than at the northern sites, and higher for PL (75.2–83.9%) and PBN (78.1–84.1%) than SBN (58.8–68.1%). Phenotypic correlations were high (> 0.5) and positive for all traits within sites (Fig. 6c); between sites, correlations were highest for PBN (77.6–85.0%) (Figure S4).

We next explored how genetic effects for the three panicle traits at the three sites varied across the genome using mash. We explored posterior summaries from mash for SNPs with the highest log10 (Bayes Factor) for genomic regions clumped at *r*^2^ ≤ 0.2 (hereafter ‘partially linked SNPs’) and regions clumped at *r*^2^ = 0 (hereafter ‘unlinked SNPs’). For partially linked SNPs, 6149 (0.23% of 2.7 M) were significant at a log10 (Bayes Factor) of 1.3; for unlinked SNPs, 146 (48.1% of 303) were significant at a log10 (Bayes Factor) of 1.3. Significant partially linked and unlinked SNPs had mash model posterior weights on covariance matrices with covariances of one across all conditions (59% and 27.1%, Fig. 6d), or high model weights on one of three data-driven matrices, two of which showed patterns of negative covariance between traits or sites (Figure S5a, b). A large fraction of posterior weight for significant partially linked and unlinked SNPs fell on the overall data-driven covariance matrix, denoted `’ED_tPCA’ (36.7% and 68.5%, Fig. 6d), which showed a pattern of positive covariances across all conditions that differed much more in comparisons of panicle trait than in comparisons across sites (Figure S5c). In addition, the median number of conditions a significant partially linked SNP affected was nine; most (59.3%) affected all nine trait-site conditions. Thus, significant SNP effects in the diversity panel data showed little evidence for GxE for panicle traits at these three gardens, supporting our QTL findings that there was little QTL × E for panicle traits across the ten common gardens.

### Enrichment tests to find candidate genes in both mapping populations

Because QTL regions were large enough that they could contain one or more effect on panicle architecture that were partially linked or even unlinked in the diversity panel, we made comparisons between QTL regions and mash SNPs using a partially linked SNP set; however, major results were similar when the full SNP set was used (data not shown). All QTL regions from the four-way cross contained significant partially linked SNPs in the diversity panel that fell within 20 kb of genes that had functionally validated roles in panicle, spikelet, or grain traits in rice (Table S4). Because many regions of the genome had significant SNPs in the diversity panel, we considered the possibility that QTL regions could be enriched with significant, partially linked SNPs from the diversity panel by chance alone. We first determined that 10 of the 18 QTL regions also had a significant enrichment of partially linked SNPs (*p* hypergeometric test < 0.05, Table S5). 0.2% of permuted genomic intervals had as many or more permuted QTL regions enriched for partially linked SNPs (*p* = 0.002, Fig. 6e), while no permuted genomic intervals had more than 10 regions significantly enriched for partially linked SNPs. Thus, even with the very different population makeup of the GWAS panel, we could confirm a higher than expected overlap between SNP effects and QTL effects on panicle traits.

Finally, we examined specific SNP effects across traits and sites for significant unlinked SNPs (146 SNPs). The alternate allele of these SNPs typically had a positive effect on panicle traits at all three sites for all three panicle traits, with the largest effects on PBN and the smallest effects for SBN, and a MAF between 0.025 and 0.075 (Fig. 6f). Rarely, antagonistic pleiotropy between CLMB and the other two sites was observed, as for the SNP at 47.94 Mb on Chr02K; effects on PL were commonly larger at PKLE than at the other two sites, which contributed to the higher phenotypic variance explained by these SNPs at this site.

We found 497 candidate genes by filtering for genes in the confidence intervals of both the QTL from the four-way population and within 20 kb of significant, partially linked SNPs from the diversity panel (Table S4). Among these overlapping candidate genes, we identified key hormone-related genes associated with panicle development. For example, a homolog of the rice DELLA protein SLR1 (Pavir.9NG141800) was found in the overlapping interval on Chr9N for both PL and PBN. SLR1 is a component involved in GA signaling pathway and regulates panicle length and branch number via the DELLA–KNOX signaling pathway (Su et al. 2021). Another candidate gene, Pavir.2KG521100, is the homolog of OsGH3.8 and was found in the overlapping interval on Chr2K for both PL and PBN. In rice, OsGH3.8 mediates cross talk between miR156-SPL7 and auxin pathways to regulate panicle architecture (Dai et al. 2018). These candidates suggest an important role for auxins and gibberellins in panicle development in switchgrass. Two flowering time genes, a homolog of rice Hd16 (Pavir.5NG232181) and a DOF transcription factor (Pavir.5NG191200), were found in the interval affecting PBN on Chr05N, which also overlaps a QTL interval for flowering in this population (Lowry et al. 2019). These genes are known to be involved in the photoperiodic flowering pathway and control panicle morphology in rice (Hori et al. 2013; Wu et al. 2015). Interestingly, Hd16 encodes a casein kinase I and phosphorylates the DELLA protein SLR1, suggesting a potential interaction between candidate genes in the overlapping intervals on Chr05N and Chr9N (Dai and Xue, 2010).

## Discussion

There has been considerable interest in the molecular mechanisms of GxE across a diversity of phenotypes, species, and environments. GxE is common and is often driven by differential sensitivity of alleles and may play an important role in adaptive plasticity and local adaptation (Des Marais et al. 2013). With its large scale, our study evaluated the genetic basis and examined the GxE of panicle morphological traits in switchgrass from a four-way mapping population which were grown at ten field sites in the central USA and from a diversity panel which were grown at three of the ten sites. Overall, we detected moderate heritability (except for the field site Stillwater, OK from the four-way) for panicle traits and positive phenotypic correlations between traits at each site for both populations. These data suggest considerable standing genetic variation in inflorescence characteristics available for natural or artificial selection to act upon. Our study identified genomic regions (QTL) that contribute to panicle trait variation across a broad latitudinal gradient. These QTL exhibited constant effects (i.e., no QTL × E: 12 QTL), antagonistic pleiotropy (i.e., sign change: 2 QTL), or site-specific effects (i.e., magnitude change: 4 QTL) across the studied environmental gradients. Most QTL with QTL × E are conditionally neutral. This is consistent with a recent meta-analyses which found that asymmetry of QTL effects are more often caused by conditional neutrality than by trade-offs (Wadgymar et al. 2017). We also did GWAS analyses and enrichment tests to find overlapping candidate genes using an independent switchgrass diversity panel, increasing our confidence in the genomic regions and candidate genes influencing panicle traits in switchgrass.

We were only able to measure panicle traits for three field sites using the diversity panel, compared to ten sites for the four-way. However, the diversity panel contains hundreds of representatives from three distinct genetic subpopulations of switchgrass, and thus captures substantially more natural variation than the four parents of the four-way, which came from two genetic subpopulations of switchgrass. In addition, we were able to obtain a balanced sample of 382 switchgrass genotypes grown at all three field sites for the diversity panel. We consider these panels complementary, and using both increases our power to distinguish true from false positives in GWAS mapping, while amplifying signals of causal QTL in the four-way that may be rare in the GWAS population (Brachi et al. 2010). In the four-way, most of the identified QTL showed no GxE effect. In the diversity panel, we found that most significant, partially linked and unlinked SNP effect patterns had high posterior weights on covariance matrices where all effects were positively correlated, either all one (60% and 27.1%) or with correlations that differed by panicle traits and field sites (ED_tPCA, 36.7% and 68.5%). These patterns of covariance corresponded to patterns of consistent, stable effects with little or no GxE across sites. Most patterns of SNP effects for unlinked SNPs were the same sign for all panicle traits and sites and similar magnitudes for the same panicle trait across sites (Fig. 6f). Thus, we found stronger evidence for pleiotropic effects on panicle traits in the diversity panel than in the four-way, and weaker evidence for effects with GxE.

GWAS analyses on panicle morphology-related traits have been conducted in other crops like rice and sorghum, either in single environment or multiple environments (i.e., different locations, different growing seasons, and/or different managements) (Zhao et al. 2016; Thapa et al. 2021; Zhong et al. 2021; Wang et al. 2021). QTL identification in GWAS with multiple environments often considers QTL detected in at least two environments as significant QTL, while rarely focusing on the effects of QTL in different environments (Ta et al. 2018; Wang et al. 2021). For example, Wang et al. (2021) considered the association to be strong when it reached the Bonferroni correction P value in at least two environments, while their experiment studying sorghum was actually grown in 11 environments. In our study, we used a more formal approach to quantify the effects across environments in the GWAS panel and found strikingly consistent effects across sites for most loci. We also completed a search for the overlapping SNPs with genomic regions identified from the four-way. After univariate GWAS, we re-estimated effects of SNPs on panicle traits while sharing information across all panicle traits and field sites using mash. Then, we conducted a permutation analysis to ask if significant SNP effects estimated using mash were enriched in QTL regions in the four-way, and if so, if the QTL regions were enriched more than random sets of genomic intervals. Ten QTL had significant enrichments of significant SNPs from the diversity panel (Table S5), more than 99.8% of random genomic intervals. In addition, we identified 6149 LD blocks within 20 kb of 497 candidate genes in regions identified by both four-way and diversity panel mapping (Table S4).

Many candidate genes for panicle traits have been reported in various crop plants and model systems (Doust 2007; Doust et al. 2005; McSteen 2006; Miura et al. 2010; Vollbrecht et al. 2005; Wang et al. 2021). In our study, candidate gene *Pavir.9NG141800,* a homolog of the rice DELLA protein SLR1, was found on Chr9N for a QTL affecting both panicle length and primary branching number. SLR1 was shown to physically interact with the meristem identity class I KNOTTED1-LIKE HOMEOBOX (KNOX) protein OSH1 to repress OSH1-mediated activation of downstream genes that are related to panicle development, providing a mechanistic link between gibberellin and panicle architecture morphogenesis (Su et al., 2021). The candidate gene Pavir.5NG191200, a homolog of rice Dof (DNA binding with one finger) transcription factor, was found on Chr05N. Wu et al. (2015) found that overexpressing *OsDof12* led to smaller panicles by decreasing primary and secondary branch numbers. They further performed the Brassinosteroid (BR)-responsive tests and found that overexpression of *OsDof12* could also result in BR hyposensitivity, suggesting that *OsDof12* is involved in rice plant architecture formation by suppressing BR signaling.

In addition to the candidates compared with rice and Arabidopsis, we also compared the candidate genes with Setaria from the study of Doust et al. (2005), in which they identified the genomic regions controlling panicle traits between foxtail millet and green millet. Seteria is a grass in the subfamily Panicoideae, the same subfamily as maize and sorghum, and closer to switchgrass than rice. We found seven corresponding candidates from the four-way and one candidate from the diversity panel, mainly for panicle length and primary branching number. These candidates are the homologs of *ba1* (*barren stalk1*), *tb1* (*teosinte branched1*), and *bif2* (*barren inflorescence2*) genes found in other crops. *ba1* represents one of the genes involved in the earliest patterning of maize inflorescences. The mutant phenotype of *ba1* causes a reduction or elimination of branches and spikelets (Gallavotti et al. 2004). *tb1* encodes a non-canonical basic helix-loop-helix protein required for the initiation of all aerial lateral meristems in maize (Studer et al. 2017), and is a key regulator of apical dominance and inflorescence architecture in bread wheat (Dixon et al. 2018). Together with the *tb1* gene, *ba1* regulates vegetative lateral meristem development (Gallavotti et al. 2004). *bif2* affects axillary meristems in the maize inflorescence. Mutants of *bif2* make fewer branches owing to a defect in branch meristem initiation, exhibiting reduced formation of all axillary structures including tassel branches, spikelets, and ear shoots (McSteen and Hake 2001). These comparisons provide more candidates for consideration. Together with other candidate genes, they might be targets for future switchgrass molecular research and breeding for panicle architecture.

In summary, our results suggest that variation of panicle traits in switchgrass is predominantly due to stable, consistent QTL that do not display GxE, with a minority of QTL displaying different effects across geographic regions (i.e., GxE). Future work focusing on the few QTL with GxE could identify rarer drivers of QTL by environment interactions in panicle traits, to help facilitate the selection of suitable genotypes of switchgrass for specific environments. Molecular research on candidate genes could provide insights to the pathways and mechanisms in panicle development in switchgrass.

## Supplemental files

The phenotyping data (panicle length, PL; primary branching number, PBN; and secondary branching number, SBN) for genotypes in the four-way at each of the ten field sites and in the diversity panel at each of the three sites (Table S1), the GWAS parameters (Table S2), the number of significant mash SNPs for each trait-site combination (Table S3), the candidate gene lists (Table S4), and the enrichment test results for diversity panel SNPs within the four-way QTL regions (Table S5) are included in the supplemental excel files. A visualization of the population structure correction in the diversity panel (Figure S1), the univariate Manhattans and QQ-plots from genome-wide association in the diversity panel (Figure S2), ecotype-specific phenotypic distributions in the diversity panel (Figure S3), phenotypic correlations between all traits and sites in the diversity panel (Figure S4), and the data-driven covariance matrices specified by mash (Figure S5) are also provided in the Supplementary Information file.

## Supplementary Information

Below is the link to the electronic supplementary material.

Supplementary file1 (XLSX 319 KB)

Supplementary file2 (XLSX 10 KB)

Supplementary file3 (XLSX 134 KB)

Supplementary file4 (CSV 106 KB)

Supplementary file5 (CSV 2 KB)

Supplementary file6 (DOCX 1109 KB)

## Data Availability

Whenever possible, plant material will be shared upon request. Source data and code to replicate these analyses are available at: https://github.com/lzhangUT/PanicleData.git. Large genetic data files to replicate these analyses are available from the UT dataverse at: *make_stable_doi_link*.
