# Supplementary material for: The genetic basis for panicle trait variation in switchgrass (Panicum virgatum)
Source: Theor Appl Genet. 2022 Jul 2;135(8):2577–92. doi: 10.1007/s00122-022-04096-x (PMC9325832; doi:10.1007/s00122-022-04096-x)
Supplement: Supplementary file 6 — Supplementary file6 (DOCX 1109 KB) [file 122_2022_4096_MOESM6_ESM.docx]

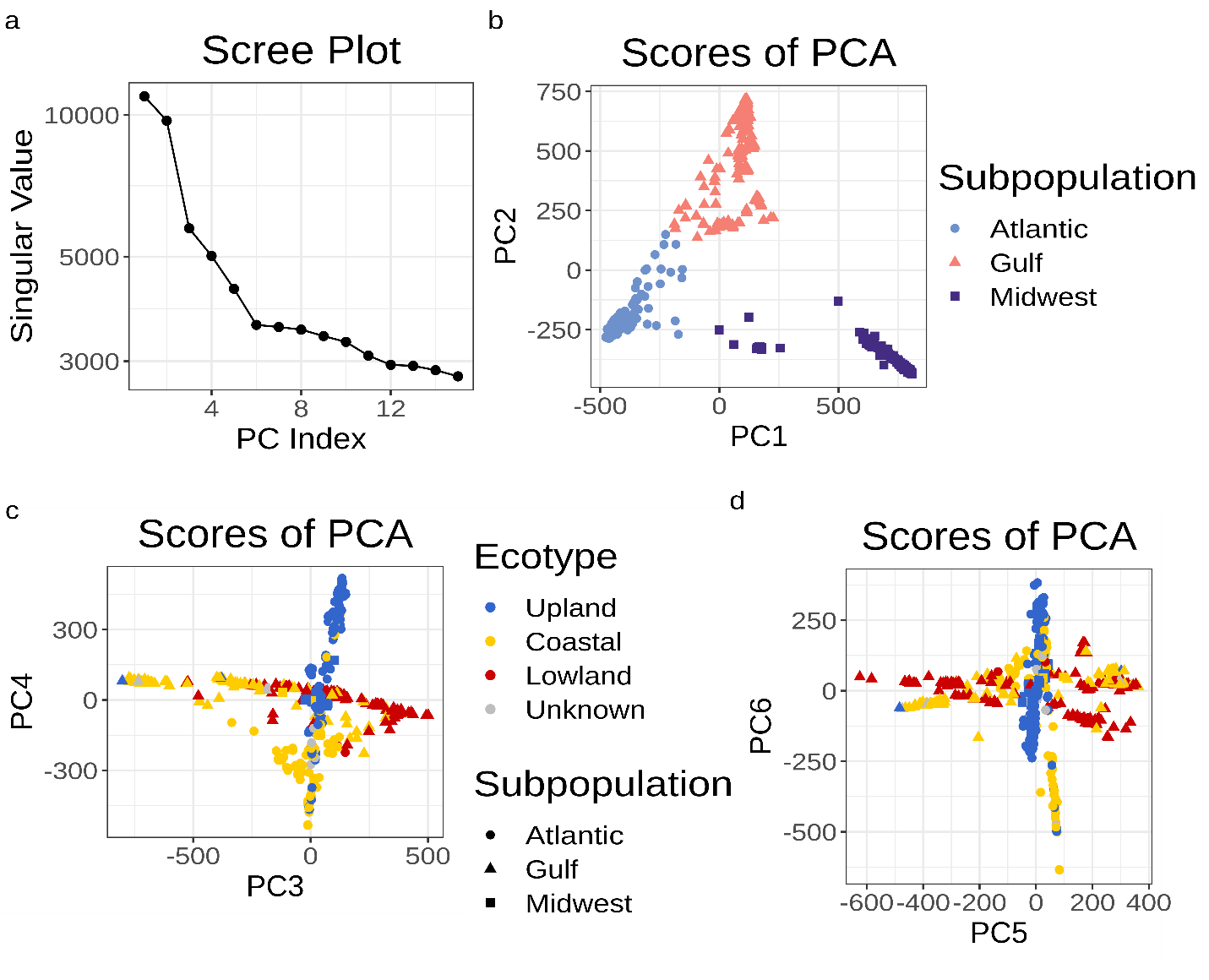


**Figure S1.** Principle components (PCs) from the singular value decomposition analysis of population structure used as covariates in the genome-wide association studies (GWAS). a) Scree plot demonstrating the amount of variation explained by each PC. b) First and second PC scores for each individual used in panicle trait GWAS. Colors represent the three genetic subpopulations identified in Lovell et al 2021. c) Third and fourth, d) fifth and sixth PC scores for each individual used in panicle trait GWAS. Colors represent the three ecotypes identified in Lovell et al 2021, while shapes represent the three genetic subpopulations.

**
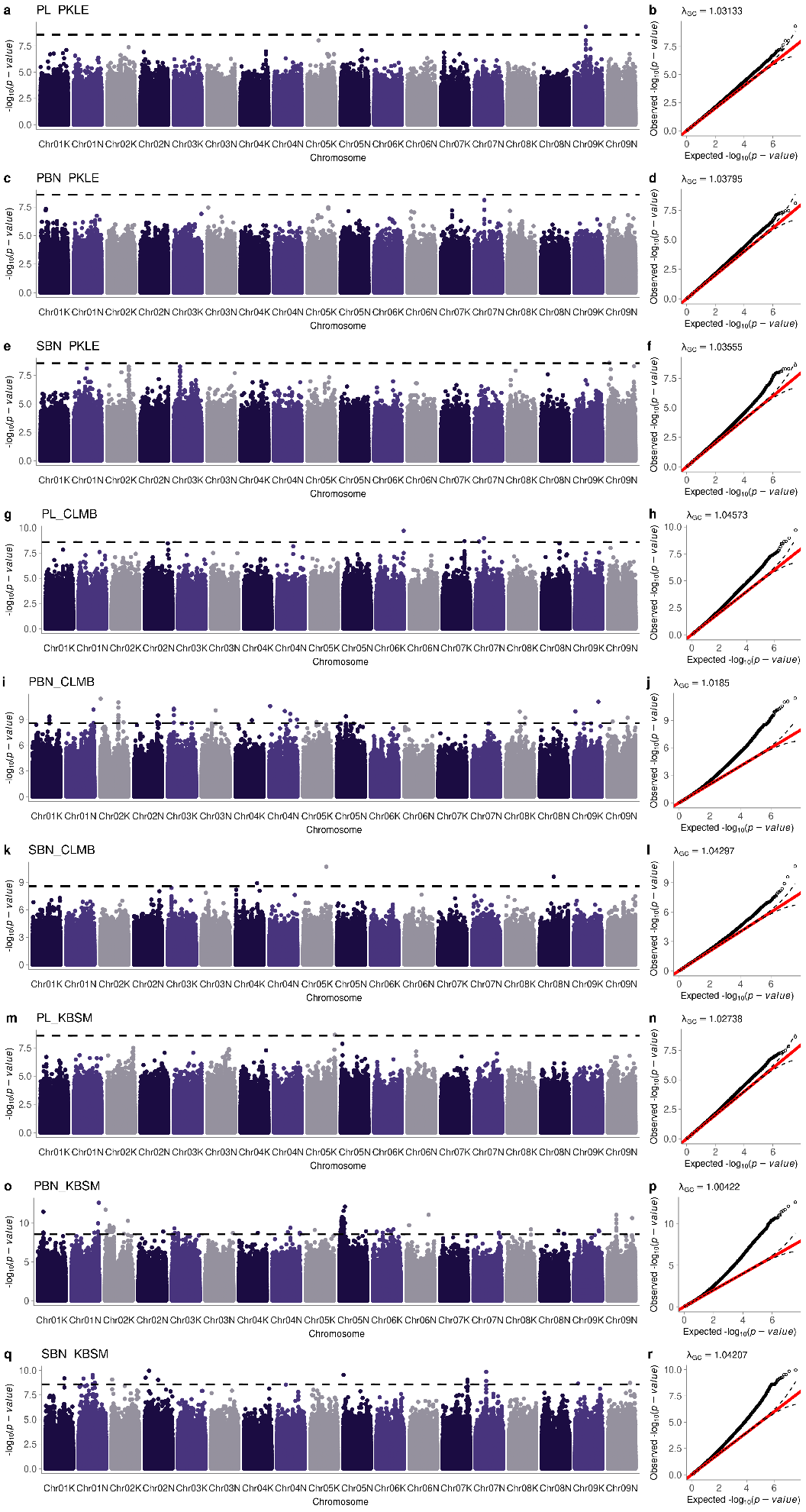
**

**Figure S2.** Manhattan and quantile-quantile plots generated for each univariate genome-wide association study run on three panicle traits (PL, panicle length; PBN, primary branch number; SBN, secondary branch number; see Figure 2a) at three common gardens (PKLE: Austin, Texas; CLMB: Columbia, Missouri; KBSM: Kellogg Biological Station, Michigan; see Figure 1a). Traits are indicated above the Manhattan plot. (a-b) PL at PKLE (c-d) PBN at PKLE (e-f) SBN at PKLE (g-h) PL at CLMB (i-j) PBN at CLMB (k-l) SBN at CLMB (m-n) PL at KBSM (o-p) PBN at KBSM (q-r) SBN at KBSM.


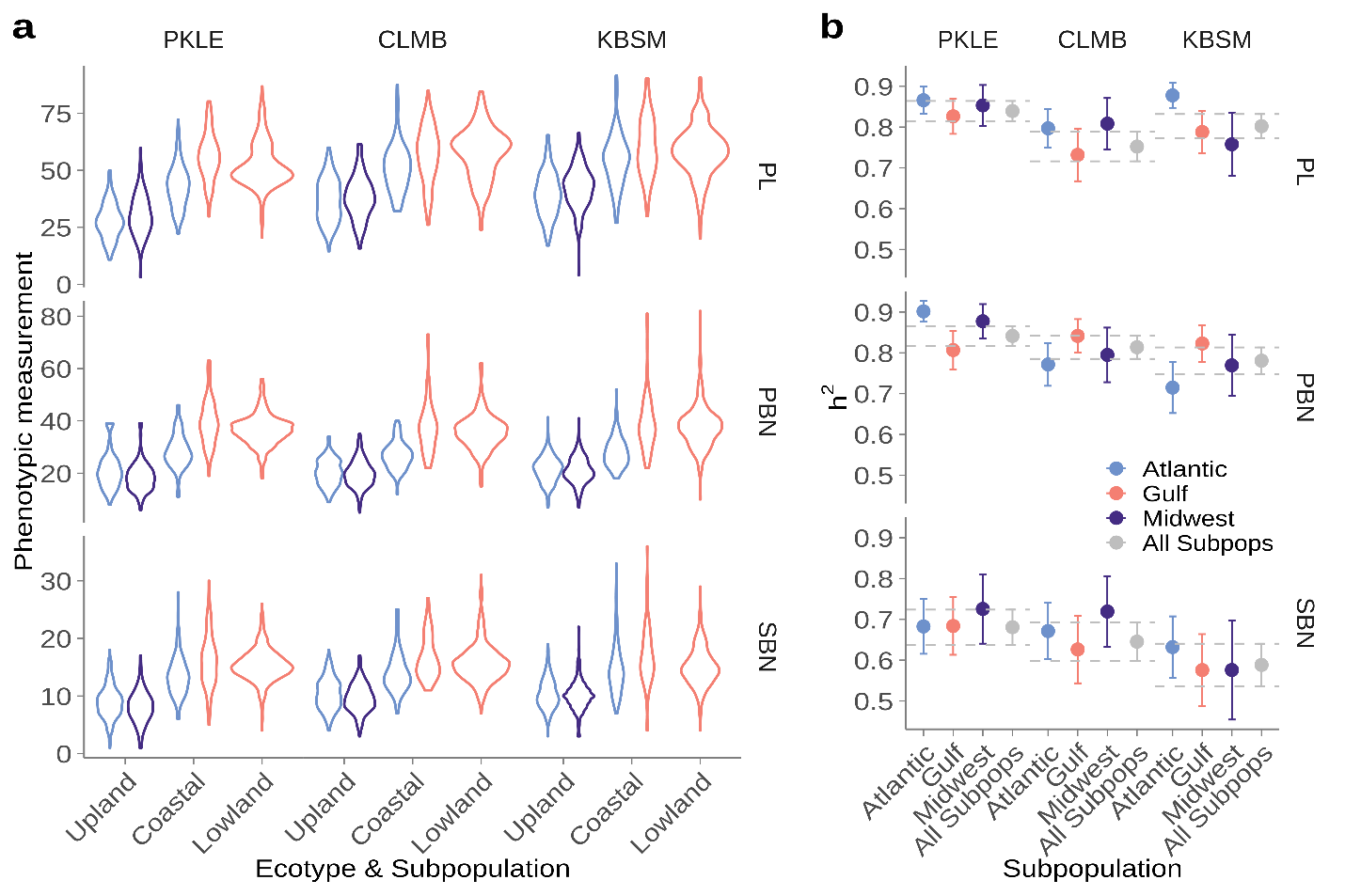


**Figure S3.** Ecotype and genetic subpopulation-specific phenotypic distributions and heritabilities for three panicle traits at three field sites. (PL, panicle length; PBN, primary branch number; SBN, secondary branch number; see Figure 2a) at three common gardens (PKLE: Austin, Texas; CLMB: Columbia, Missouri; KBSM: Kellogg Biological Station, Michigan; see Figure 1a).Colors represent the three genetic subpopulations identified in Lovell et al 2021. a) The phenotypic distribution of the diversity panel (violin plots) separated by ecotype and genetic subpopulation (colors). b) Narrow-sense heritability (*h*^2^) estimates for each panicle trait estimated separately for each genetic subpopulation at each site. Error bars represent two times the standard error. Dashed lines represent the range of the heritability estimates for panicle traits estimated jointly for all genetic subpopulations at each site.


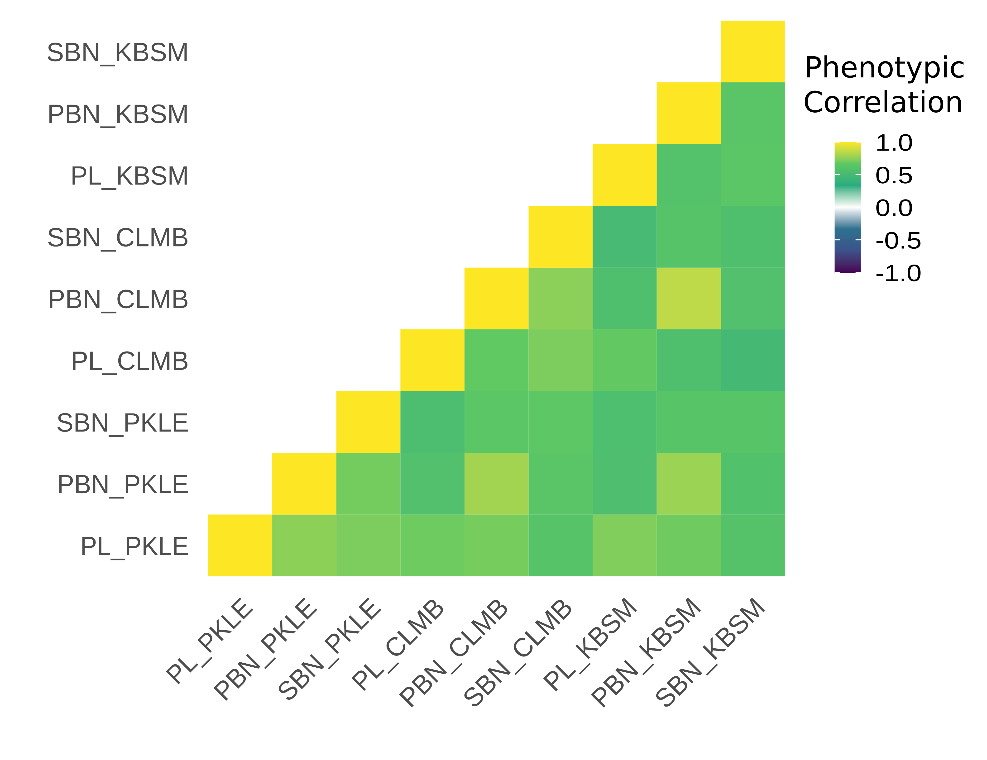


**Figure S4.** Phenotypic correlations in the diversity panel across all three panicle traits expressed at all three field sites. PL, panicle length; PBN, primary branch number; SBN, secondary branch number; see also Figure 2a. PKLE: Austin, Texas; CLMB: Columbia, Missouri; KBSM: Kellogg Biological Station, Michigan; see also Figure 1a.


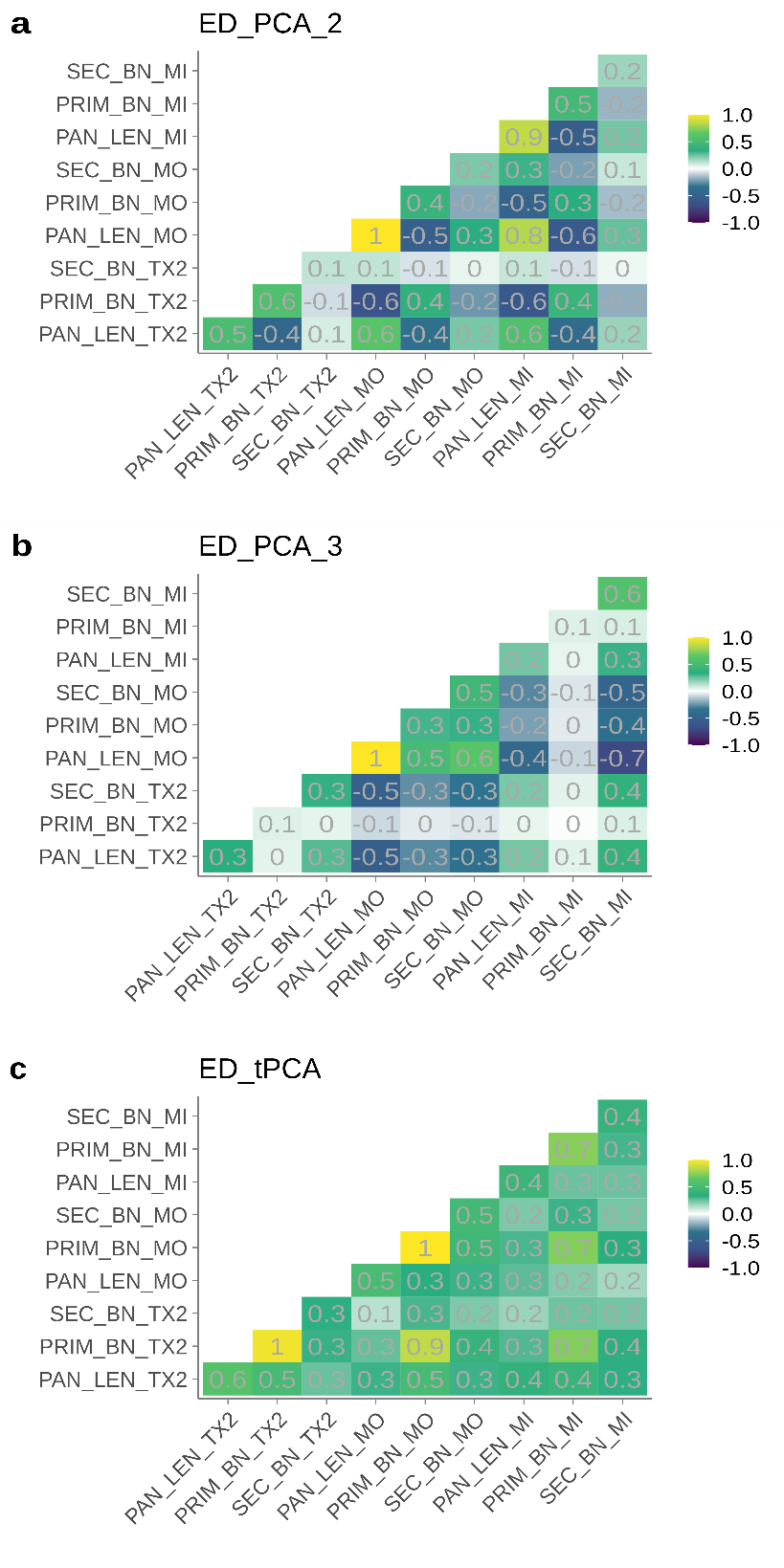


**Figure S5.** Data-driven covariance matrices specified using multivariate adaptive shrinkage in the diversity panel across all three panicle traits expressed at all three field sites. Colors represent correlations between pairs of trait by environment combinations. See Figure 6d for the posterior weights on each of these matrices summed across all 18.7M SNPs. PL or PAN_LEN, panicle length; PBN or PRIM_BN, primary branch number; SBN or SEC_BN, secondary branch number; see also Figure 2a. PKLE or TX2: Austin, Texas; CLMB or MO: Columbia, Missouri; KBSM or MI: Kellogg Biological Station, Michigan; see also Figure 1a.
